# Predictors of mental health problems during the COVID-19 outbreak in Egypt in 2021

**DOI:** 10.3389/fpubh.2023.1234201

**Published:** 2023-11-09

**Authors:** Suzan Abdel-Rahman, Fuad A. Awwad, Emad A. A. Ismail, B. M. Golam Kibria, Mohamed R. Abonazel

**Affiliations:** ^1^Department of Demography and Biostatistics, Faculty of Graduate Studies for Statistical Research, Cairo University, Giza, Egypt; ^2^Department of Quantitative Analysis, College of Business Administration, King Saud University, P.O. Box 71115, Riyadh 11587, Saudi Arabia; ^3^Department of Mathematics and Statistics, Florida International University, Miami, FL, United States; ^4^Department of Applied Statistics and Econometrics, Faculty of Graduate Studies for Statistical Research, Cairo University, Giza, Egypt

**Keywords:** COVID-19, Egypt, food insecurity, LASSO, mental Health, ridge regression, social distancing

## Abstract

**Background:**

With the widespread outbreak of the coronavirus (COVID-19) pandemic, many countries, including Egypt, have tried to restrict the virus by applying social distancing and precautionary measures. Understanding the impact of COVID-19-induced risks and social distancing measures on individuals' mental health will help mitigate the negative effects of crises by developing appropriate mental health services. This study aimed to investigate the most contributing factors that affected individuals' mental health and how individuals' mental health has changed over the lockdown period in Egypt in 2021.

**Methods:**

The study draws on a nationally representative sample from the combined COVID-19 MENA Monitor Household Survey conducted by the Economic Research Forum. The data were collected in Egypt by phone over two waves in February 2021 and June 2021. The total number of respondents is 4,007 individuals. The target population is mobile phone owners aged 18–64 years. The 5-item World Health Organization Well-Being Index (WHO-5) is used to assess the individuals' mental health over the past 2 weeks during the pandemic. Penalized models (ridge and LASSO regressions) are used to identify the key drivers of mental health status during the COVID-19 pandemic.

**Results:**

The mean value of mental health (MH) scores is 10.06 (95% CI: 9.90–10.23). The average MH score for men was significantly higher than for women by 0.87. Rural residents also had significantly higher MH scores than their urban counterparts (10.25 vs. 9.85). Middle-aged adults, the unemployed, and respondents in low-income households experienced the lowest MH scores (9.83, 9.29, and 9.23, respectively). Individuals' mental health has deteriorated due to the negative impacts of the COVID-19 pandemic. Regression analysis demonstrated that experiencing food insecurity and a decrease in household income were independent influencing factors for individuals' mental health (*p* < 0.001). Furthermore, anxiety about economic status and worrying about contracting the virus had greater negative impacts on mental health scores (*p* < 0.001). In addition, women, middle-aged adults, urban residents, and those belonging to low-income households were at increased risk of poor mental health (*p* < 0.05).

**Conclusion:**

The findings reveal the importance of providing mental health services to support these vulnerable groups during crises and activating social protection policies to protect their food security, incomes, and livelihoods. A gendered policy response to the pandemic is also required to address the mental pressures incurred by women.

## 1. Introduction

In response to the coronavirus (COVID-19) outbreak, most governments have made great efforts to sustain healthcare services and minimize the risk of the pandemic consistent with international guidelines of the World Health Organization (WHO). Various strategies have been implemented with different degrees of success in containing the pandemic (1, 2). Egypt has taken precautionary measures to hinder its spread such as approval of a presidential decree declaring a nationwide state of emergency, regional lockdowns, suspending flights, closures of schools, nurseries, and childcare homes, canceling community events, and reducing working hours. Egypt has also imposed a nationwide curfew, suspended public transportation, banned public gatherings, implemented quarantine, and other social distancing measures (3).

Precautionary measures have been associated with rapid and profound implications on individuals' mental health. Recent studies have documented an increase in symptoms of depression and anxiety due to lifestyle changes induced by the pandemic (4, 5). Lockdown measures have led to disruptions in working hours, physical activity, sleep habits, time use, and social interactions. Social distancing, confinement at home, illness, and the death of relatives contributed to depression and mental disorders. Furthermore, the negative labor market outcomes such as loss of job and income have worsened individuals' mental health (6).

Particular groups have been disproportionately affected by the COVID-19 crisis. Younger age groups have faced multiple economic shocks during the pandemic, and their well-being was more negatively affected than older age groups (7, 8). Women were also among the vulnerable groups exposed to the negative effects of the pandemic. They were overrepresented in the affected sectors, such as food and accommodation, health and social work, travel, and labor-intensive industrial activities (9). Moreover, the increased childcare responsibilities due to the closure of schools and childcare homes played a significant role in declining income and reducing the labor supply for working mothers (10, 11). These burdens likely put women at greater risk of physical and mental health (12, 13). Medical students and healthcare workers were also more likely to suffer psychological disturbances during the pandemic, showing moderate-to-severe stress, anxiety, and depressive symptoms, respectively (14–17).

Numerous studies have evaluated the effects of lockdown policies on mental health and have well-documented the positive association between lockdown measures and mental disorders, including depression, anxiety, and stress. Nkire et al. (4) measured the determinants and impacts of applying self-isolation during the pandemic in Canada and found that it was significantly associated with moderate-to-high stress, anxiety symptoms, and major depressive symptoms, especially among the older adults. Möhring et al. (5) assessed the effects of working from home, reducing working hours, and closing schools and childcare homes on individuals' satisfaction with work and family life and found a pronounced decrease in work satisfaction among mothers and childless persons. Giuntella et al. (6) found that declines in physical activity and changes in lifestyle behaviors among college students were associated with higher rates of depression. Brooks et al. (18) emphasized that the COVID-19 pandemic has been associated with stress symptoms, anger, and confusion, indicating that the higher morbidity and mortality rates, income loss, and fear of stigma were the risk factors for negative mental health outcomes during the pandemic. Saikia et al. (19) found that the pandemic significantly worsened the well-being of women, low income, and younger in South Australia. In addition, Donnelly and Farina (20) found the odds of depression were greater for women, less-educated, unmarried, and younger adults.

On the other hand, some studies focused on the impact of unemployment on mental health during the pandemic. Mass layoffs and business closures due to the COVID-19 pandemic have caused profound impacts on individuals' livelihoods (21). The COVID-19 outbreak coincided with an unprecedented rise in unemployment and economic losses, affecting psychological well-being. In this context, Cotofan et al. (8) assessed the relationship between unemployment and subjective well-being and found that unemployed and inactive individuals were less satisfied with their lives than employed individuals during the pandemic. Giovanis and Ozdamar (22) estimated the well-being costs associated with coping strategies and the required money to compensate individuals who experience job and income losses. Their study found that borrowing from others and selling assets have the highest well-being costs.

The effects of COVID-19 in Arab countries are becoming increasingly documented in research (11, 23). Arafa et al. (14) estimated the prevalence rates of depression, anxiety, stress, and sleep disorder during the pandemic outbreak in four Egyptian governorates and found that women, workers outside the health sector, those who watched/read COVID-19 news, and those lacked emotional support were positively associated with severe psychological disturbances. Elkholy et al. (24) measured the mental health indicators of Egyptian healthcare workers and estimated the potential risk factors, highlighting that female healthcare workers were more likely to suffer severe depression, stress, and anxiety. AboKresha et al. (25) investigated the impact of isolation measures associated with the pandemic on violence against children in Egypt, and their study indicated that children reported a moderate-to-severe psychological impact due to increased risk of violence during the pandemic. El-Zoghby et al. (26) assessed the impact of COVID-19 on mental health and social support. Their study targeted Egyptian adults using an online questionnaire. They found that more than half of the respondents experienced increased household and financial difficulties, and more than one-third of the respondents witnessed a severe psychological impact.

Some aspects have not been adequately addressed in the previous studies in Arab countries. In Egypt, like other countries, individuals are asked to comply with stay-at-home orders, quarantine, and isolation to reduce the risk of infection and protect community health. But despite that, little evidence exists on how COVID-19 implications including risks, social distancing measures, food insecurity, and income falls have affected individuals' mental health. In this context, the current study seeks to measure the determinants of mental health during the pandemic period, with a special focus on the extent to which social distancing measures and other negative repercussions induced by the pandemic have affected mental health outcomes in Egypt.

Lockdown measures and fear of catching COVID-19 put individuals under great psychological pressure and exacerbated symptoms of stress, anxiety, and depression (27). The growing risk of mental illness caused by COVID-19 has caught the attention of the Egyptian government. The General Secretariat of Mental Health and Addiction Treatment (GSMHAT), affiliated with the Egyptian Ministry of Health and Population, launched a national mental health strategy during the pandemic. GSMHAT provided mental health psychosocial support services for all affected individuals. GSMHAT formed a national group to coordinate between governmental and non-governmental sectors, to provide mental health services. GSMHAT's five task force groups emerged to quickly respond to emergencies and other priority areas. Appendix Figure A1 illustrates the functions of the five groups. In addition, GSMHAT has activated hotline services and online therapy sessions to provide psychosocial and psychiatric services to all segments of the population. Among patients seeking consultations, 25% had anxiety symptoms, 22% had depression, and 15% reported insomnia (28).

## 2. Materials and methods

### 2.1. Data source

The study draws on a nationally representative sample from the Combined COVID-19 MENA Monitor Household Survey (CCMMHH). CCMMHH survey is conducted by the Economic Research Forum (ERF) and targeted mobile phone users aged 18–64 years using short panel surveys. The ERF collected the data over two waves in February 2021 and June 2021. The sample was selected using random digit dialing with a maximum of three attempts to fill out the questionnaire. At least 2,000 unique individuals were recruited in each wave. Individual weights were used during the analysis to delve into the extensive details of the sampling method, response rates, data collection notes, weights, survey design, and the various sections of the questionnaire, see (29).

#### 2.1.1. Mental health score

Our primary outcome is the individuals' mental health status. The survey used the 5-item World Health Organization Well-Being Index (WHO-5), a short questionnaire, to assess the individuals' mental health over the past 2 weeks during the pandemic. Individuals were asked five questions to express their subjective well-being. Using a scale ranging from 0 (at no time) to 5 (all the time), individuals rated the following statements: “I have felt cheerful and in good spirits.”, “I have felt calm and relaxed.”, “I have felt active and vigorous.”, “I woke up feeling fresh and rested.”, and “My daily life has been filled with things that interest me.” Responses to the five statements (WHO-5) are aggregated to provide an overall assessment of mental health status on a scale from 0 to 25 where 0 represents the worst mental health and 25 represents the best mental health.

#### 2.1.2. Sociodemographic characteristics

Sociodemographic characteristics are responsible for developing mental health status as demonstrated by previous studies. The explanatory variables included sociodemographic characteristics, including, sex (male/female), place of residence (rural/urban); age [younger adults (<30), adults (30–59), and older adults (≥60)]; education level (less than basic education, basic education, secondary education, and higher education); marital status (never married, currently married, and divorced or widowed); income quartiles (four quartiles where the lower quartile is denoted as the 1st quartile and the highest quartile is denoted as the 4th quartile); employment status (employed, unemployed, and out of labor force); household size; and the number of children under age 6 years living in the same household.

#### 2.1.3. Negative implications of COVID-19

We also seek to measure the impact of negative changes of COVID-19 on mental health status, including social distancing, food insecurity, risks, and a decrease in household income. Social distancing is measured by three binary variables indicating whether the individual did the action (staying at least 1 m away from others, wearing a mask outside the home, and washing hands more than before the pandemic). Food insecurity is a binary variable indicating whether the individual was food insecure during the past 7 days. Individuals were classified as food insecure if they experienced any of the following challenges: difficulty in going to food markets, inability to buy the usual amount of food because of shortages of food in markets or increase in food prices or drop in household income. COVID risks were measured using two variables: how worried individuals were about contracting COVID-19 and how worried they were about the economic situation. Individuals reported their worry on a scale from 1 to 4 (not at all worried, a little worried, rather worried, and very worried, respectively). A decrease in household income is a binary variable indicating whether the household income decreased last month compared to February 2020.

### 2.2. Statistical analysis

The frequency distribution and descriptive statistics were used to describe the characteristics of respondents. The independent samples *t*-test is used to determine whether the mean mental health score differs significantly across two groups, and analysis of variance (ANOVA) is used to compare the means of more than two groups. Statistical analysis was performed using the R program. As we have many independent variables and most are categorical variables, we used feature selection to identify the most contributing variables and exclude irrelevant variables so that we can avoid complexity in the resulting model. The shrinking approach performs variable selection and reduces efficiently the number of independent variables. It constrains the coefficients, setting the corresponding coefficient estimates to be exactly zero. Shrinking or penalized models produce more interpretable models and improve the fit by reducing the variance. We used two techniques: Ridge regression and the least absolute shrinkage and selection operator (LASSO) (30, 31). We used the “glmnet” R-package to perform the ridge and LASSO regressions.

#### 2.2.1. Ridge regression

Ridge regression is an extension of linear regression. Ride regression estimates the coefficients by minimizing a slightly different quantity. Ride regression modifies the loss function by adding a penalty parameter to minimize the model's complexity. Ridge regression seeks to estimate coefficients that fit the data by minimizing the residual sum of squares (RSS) as the least squares procedure. However, ridge regression has a term of shrinkage penalty that will be small when is close to zero. The tuning parameter (λ) controls the relative effects of the two terms (RSS and shrinkage penalty) on the coefficient estimates. A widely used cross-validation procedure is used to select the best value of λ that achieves the smallest mean squared error (MSE).

#### 2.2.2. LASSO regression

LASSO regression provides an obvious advantage of getting rid of the weakly influential variables. While ridge regression keeps all predictors in the final model as λ shrinks the coefficients without setting any of them equal to zero, LASSO generates more interpretable models by shrinking the estimates to zero when the tuning parameter λ is sufficiently large. The cross-validation error is computed for a grid of λ values and the one with the smallest cross-validation error is selected. The model is refit using all variables and the best value of λ (32). It is worth mentioning that neither the LASSO nor the ridge regression dominates the other. LASSO performs better if a few variables have substantial effects and other variables have small effects near zero, while ridge performs better if all variables contribute relatively equally to the response variable. As the relationship between predictors and the dependent variable is not known priori, cross-validation is used to determine the best approach. In this context, we apply ridge and LASSO regressions and compare them. Ridge regression gives us initial insight into which variables are weakly correlated with the outcome variable and provides their shrinking estimates, whereas LASSO regression enables the exclusion of irrelevant variables, maintaining only influential ones. The ridge model is performed over a grid of several values for λ, ranging from (10^10^ to 10^−2^). There is a vector of coefficients associated with each value of λ. The data set is split into two sets (training and test sets) to estimate the test error. A 10-fold cross-validation procedure is used to determine the optimal value of λ that has the minimum mean squared error (MSE). Ridge regression is fitted on the training data and MSE is estimated on the test data. Then, the coefficients of the ridge regression are estimated on the full data set using the optimal value of λ.

## 3. Results

### 3.1. Sociodemographic characteristics of the study respondents and mental health scores

The total number of respondents is 4,007 individuals, distributed as 2,000 individuals surveyed in February 2021 and 2007 individuals surveyed in June 2021. Overall, 63.5% of respondents are men, 62.1% are middle-aged adults (30–59 years), and over half of them (51.8%) are urban dwellers. Among all respondents, 71.5% are currently married, 51.8% are employed, and 46.5% completed secondary education. The average household size is 5 individuals. Table 1 provides descriptive statistics of other characteristics.

**Table 1:** Sociodemographic characteristics of the respondents and their mental health scores.

**Sociodemographic characteristics**	***n* (%)**	**Average mental health score and (95% CI)**	**Test statistic and *P*-value**
**Sex**			
Male	2,545 (63.5)	10.38 (10.17–10.59)	*t* = 5.09^***^ < 0.001
Female	1,462 (36.5)	9.51 (9.27–9.76)	
**Place of residence**			
Urban	2,077 (51.8)	9.85 (9.63–10.08)	*t* = 2.59^**^ 0.009
Rural	1,930 (48.2)	10.29 (10.05–10.51)	
**Age group**			
Young adults (< 30)	1,379 (34.4)	10.36 (10.09–10.63)	*F* = 8.34^***^ < 0.001
Middle-aged adults (30–59)	2,487 (62.1)	9.83 (9.62–10.04)	
Older adults (≥60)	141 (3.5)	11.23 (10.15–12.31)	
**Marital status**			
Never married	963 (24.0)	10.46 (10.14–10.79)	*F* = 4.64^**^ 0.01
Currently married	2,866 (71.5)	9.96 (9.78–10.16)	
Widowed/divorced	178 (4.4)	9.42 (8.58–10.27)	
**Education level**			
Less than basic	687 (17.1)	10.00 (9.58–10.42)	*F* =1.48 0.217
Basic	507 (12.7)	9.63 (9.21–10.26)	
Secondary	1,866 (46.6)	10.15 (9.92–10.39)	
Higher	947 (23.6)	10.16 (9.83–10.49)	
**Income quartiles**			
1st quartile	1,197 (29.9)	9.23 (8.95–9.51)	*F* =15.57^***^ < 0.001
2nd quartile	1,085 (27.1)	9.97 (9.68–1027)	
3rd quartile	938 (23.4)	10.26 (9.91–10.59)	
4th quartile	454 (11.3)	11.35 (10.83–11.87)	
**Employment status**			
Employed	2,328 (58.1)	10.39 (10.17–10.60)	*F* = 13.69^***^ < 0.001
Unemployed	831 (20.7)	9.29 (8.97–9.61)	
Out of labor force	484 (21.2)	9.93 (9.57–10.29)	
**Number of children**			
**0**	2,064 (51.5)	10.15 (9.92–10.38)	*F* = 1.03 0.357
1–2	1,765 (44.1)	10.00 (9.77–10.24)	
3+	178 (4.4)	9.62 (8.82–10.42)	
**Total**	4,007	10.06 (9.90–10.26)	

^***^Refers to highly significant results at a P-value of < 0.001.

^**^Refers to very significant results at a P-value of < 0.01.

Individuals reported their various feelings and related frequencies during the last 2 weeks. The results show that the percentage of respondents who felt cheerful and in good spirits most of the time did not exceed 12% and some of the time was 37%. Moreover, 18% of respondents did not feel calm and relaxed in the past 2 weeks and only 7.3% felt calm and relaxed more than half of the time. More than one-third of respondents (33.3%) were active and vigorous some of the time and 12% all the time. Almost one-quarter of respondents reported that their daily lives were full of things that interest them all the time during the past 2 weeks. Other responses are given in detail in Appendix Table A. Responses to these five questions are aggregated and provide an overall assessment of mental health status with a mean value of 10.06 points (95% CI: 9.90–10.23).

The average mental health score (MH) for men was significantly higher than for women by 0.87 points. Rural residents also experienced significantly higher MH scores than their urban counterparts. Middle-aged adults had the lowest MH score (9.83 points) while the older adults had the highest score (11.23 points). Divorced and widowed respondents showed lower MH scores than the never-married and currently married respondents. The unemployed had lower MH scores compared to employed or individuals outside the labor force. The higher the household income level, the higher the average mental health score of respondents. There were no significant differences in MH scores by educational level or number of children.

The gender gap in mental health scores varied according to age. Young men experienced higher MH scores than their women counterparts by 0.96 points. The gender gap increased in the older adults category by 2.3 points in favor of men, while converged in the middle-aged group. The gender gap in mental health scores did not minimize as the education level increased. The MH scores for women and men are almost equal at different levels of education. The results also highlight that the average MH score of employed individuals during the pandemic was higher than that of the unemployed and economically inactive individuals regardless of the household income quartile.

### 3.2. Negative implications of COVID-19 and corresponding mental health scores

Table 2 indicates that increasing individuals' anxiety about their economic situation is accompanied by a significant decline in mental health scores. Very worried individuals had the lowest MH score compared to those who were not at all worried (8.93 points vs. 12.06 points). In the same vein, the higher individuals' anxiety about contracting the virus, the lower their mental health. Individuals who have contracted COVID-19 also experienced lower mental health (8.79 points). However, social isolation and adherence to social distancing measures are expected to worsen the mental health status. There were insignificant differences between individuals who adhered to social distancing measures and those who did not. Experiencing food insecurity exacerbated mental health, individuals who experienced food insecurity had lower MH scores than those who did not (9.21 points vs. 11.79 points). Economic loss and food insecurity are interconnected and cause mental health disorders. Individuals who reported that their household income decreased due to the pandemic experienced poor mental health (9.19 points).

**Table 2:** Distribution of study respondents according to COVID-19 implications and their mental health scores in Egypt.

**COVID-19 implications**	***n* (%)**	**Average mental health score and (95% CI)**	**Test statistic and *P*-value**
**Risks**			
**Worrying about economic situation**			
Not at all worried	795 (19.8)	12.06 (11.62–12.5)	*F* =73.18^***^ < 0.001
A little worried	666 (16.6)	10.61 (10.24–10.98)	
Rather worried	774 (19.3)	10.14 (9.82–10.47)	
Very worried	1,772 (44.2)	8.93 (8.71–9.14)	
**Worrying about catching COVID-19**			
Not at all worried	1,428 (35.6)	10.83 (10.52–11.14)	*F* = 20.03^***^ < 0.001
A little worried	608 (15.2)	10.42 (10.05–10.78)	
Rather worried	812 (20.3)	9.89 (9.57–10.20)	
Very worried	1,024 (25.6)	9.09 (8.79–9.38)	
I had it already	135 (3.4)	8.79 (8.01–9.55)	
**Social distancing**			
**Staying at least 1 m away from people**			
Yes	3,466 (86.5)	10.07 (9.89–10.23)	*t* =0.129 0.897
No	541 (13.5)	10.04 (9.55–10.52)	
**Wearing masks outside the house**			
Yes	3,545 (88.5)	10.11 (9.94–10.28)	*t* = 1.55 0.114
No	462 (11.5)	9.70 (9.22–10.19)	
**Wishing hands more often than before COVID-19**			
Yes	3,460 (86.3)	10.09 (9.92–10.26)	*t* = 0.723 0.470
No	547 (13.7)	9.91 (9.43–10.39)	
**Household food insecurity**			
Yes	2,688 (67.1)	9.21 (9.04–9.39)	*t* = 15.08^***^ < 0.001
No	1,319 (32.9)	11.79 (11.49–12.09)	
**Decrease in household income**			
Yes	1,848 (46.1)	9.19 (8.97–9.40)	*t* = 9.94^***^ < 0.001
No	2,159 (53.9)	10.81 (10.58–11.04)	

^***^P-value < 0.001.

### 3.3. Predictors of mental health scores during the pandemic

Ridge regression and LASSO regressions are used to determine the most contributing variables. The coefficients of the ridge regression are estimated on the full data set using the optimal value of λ = 0.798 which corresponds to the smallest MSE (24.38). LASSO regression is also estimated using the best value for λ = 0.069. The effects of some variables became larger in magnitudes in the LASSO regression, indicating their relative importance in predicting mental health. LASSO regression did not outperform the ridge regression and produced a similar MSE (25.79) but yielded a more accurate and interpretable model.

Ridge and LASSO regressions highlighted the role of sociodemographic variables in influencing mental health status during the pandemic. As shown in Table 3, the mental health status of women, urban dwellers, and middle-aged adults (30–59) was significantly worse than that of men, rural dwellers, and young adults (<30). The higher the household income level, the higher the individual's mental health score, with individuals in the fourth income quartile exhibiting greater mental health scores than individuals in the first income quartile by 1.27 points. Unemployed respondents were severely affected by COVID-19 and had lower mental health scores than employed. The unexpected finding was the negative association between the education level and mental health scores where highly educated individuals had lower mental health scores than those with less than basic education. While the number of children, household size, and marital status had little and insignificant impacts on individuals' mental health in ridge regression, and their effects faded in the LASSO regression. Anxiety about the economic situation had a greater impact on the mental health score with a decrease of more than two points among very worried respondents in both ridge and LASSO regressions. Worrying about contracting COVID-19 was also associated with lower levels of mental health. Experiencing food insecurity and reduced household income due to the COVID-19 pandemic have retained their importance in explaining individuals' mental health in both ridge and LASSO regressions and were significantly associated with poor mental status. Adherence to social distancing measures through wearing a mask outside the home and constantly washing hands was positively associated with mental health scores, while staying at least 1 m away from others had no significant effect. No significant difference was found between the (February 2021) wave and the (June 2021) wave.

**Table 3:** Estimates of the ridge and LASSO regression for mental health status during the pandemic.

**Variables^a^**	**Ridge regression**	**LASSO regression^b^**
	**Coefficient (SE)**	**Coefficient (SE)**
**Sociodemographic variables**		
Female	−0.338^*^ (0.178)	−0.842^**^ (0.320)
Urban	−0.509^**^ (0.163)	−0.606^*^ (0.284)
**Age group**		
Middle-aged adults (30–59)	−0.449^*^ (0.215)	−0.599^*^ (0.125)
Older adults (≥60)	0.254 (0.484)	——
**Marital status**		
Currently married	−0.065 (0.272)	——-
Widowed/divorced	−0.238 (0.462)	——-
**Education level**		
Basic	−0.547^+^ (0.293)	−0.811 (0.462)
Secondary	−0.228 (0.231)	−0.184 (0.246)
Higher	−0.669^*^ (0.272)	−1.007^+^ (0.535)
**Income quartiles**		
2nd quartile	0.599^**^ (0.214)	0.689^**^ (0.284)
3rd quartile	0.614^**^ (0.231)	0.814^**^ (0.241)
4th quartile	1.273^***^ (0.302)	1.298^***^ (0.342)
**Employment status**		
Unemployed	−0.589^*^ (0.227)	−0.522^*^ (0.241)
Out of labor force	−0.373 (0.245)	——-
**Number of children**		
1–2	−0.090 (0.349)	——-
3+	−0.626 (0.837)	−0.724 (0.871)
**Household size**	−0.060 (0.048)	——
**Household food insecurity**	−1.685^***^ (0.184)	−2.012^***^ (0.294)
**Decrease in household income**	−0.828^***^ (0.166)	−0.929^***^ (0.162)
**Risks**		
**Worrying about economic situation**		
A little worried	−1.264^***^ (0.271)	−1.291^***^ (0.231)
Rather worried	−1.436^***^ (0.265)	−1.483^***^ (0.264)
Very worried	−2.141^***^ (0.235)	−2.196^***^ (0.233)
**Worrying about catching COVID-19**		
A little worried	−0.016 (0.251)	——
Rather worried	−0.597^*^ (0.235)	−0.599^**^ (0.232)
Very worried	−0.808^***^ (0.228)	−0.809^***^ (0.227)
I had it already	−1.624^***^ (0.454)	−1.695^***^ (0.453)
**Social distancing**		
Staying at least 1 m away from people	0.296 (0.249)	—–
Wearing masks outside the house	0.547^*^ (0.266)	0.598^*^ (0.244)
Wishing hands more often than before COVID-19	0.561^*^ (0.245)	—–
**Wave (June 2021)**	−0.135 (0.157)	——
**MSE**	24.38	25.79
**Adjusted R-squared**	0.103	0.106

^a^The reference groups for demographic social variables are male, younger adults (< 30), rural, never married, less than basic education, the first quartile, not having children, not at all worried, and wave (Feb. 2021).

^***^P-value < 0.001.

^**^P-value < 0.01.

^*^P-value < 0.05.

^+^p < 0.1.

^b^(—-) means that the resulting coefficient from LASSO regression is exactly zero.

## 4. Discussion

This study is among the few Egyptian studies that investigated the impact of the pandemic on mental health. It examined the impact of COVID-19-induced risks and social distancing measures on individuals' mental health using the latest COVID-19 data available for Egypt by the Economic Research Forum. The study also highlighted key differences in the individuals' mental health by sociodemographic characteristics, including gender, age, income quartile, educational level, employment status, and place of residence, and investigated the significant drivers of mental health outcomes during the pandemic. The current study showed that anxiety about the economic situation and catching COVID-19 infection were the core drivers of mental health during the pandemic period. Experiencing food insecurity and a decline in household income contributed to mental health deterioration. In addition, sociodemographic variables have obvious effects on mental health status during the pandemic.

The current study found that women were more likely to have a poor mental health status than men. These findings are in line with other studies which indicated that women suffered from psychological disturbances and lower levels of well-being more than men during the pandemic (14, 19, 20, 33). For several reasons, women are expected to suffer poor mental health during the pandemic. On one hand, there is a large gender gap in childcare and house responsibilities in developing countries, where women bear the largest burden regardless of their husbands' work arrangements, and the situation is expected to worsen during the pandemic. Closures of schools and daycare facilities have destabilized the daily life of working women, pushing them to devote more time to childcare and homeschooling during the COVID-19 period. Barsoum and Majbouri (13) found that COVID-19 has negatively affected women's well-being due to increased unpaid care work and domestic workloads, and more than one-third of Arab women reported spending more hours caring for children and doing household chores during the pandemic than before (34). Women are more likely to experience anxiety, depression, and poor mental health during the pandemic because they are the family's primary caregivers and are more concerned about their families' health during the pandemic outbreak. On the other hand, women are more concentrated in labor-intensive industries that have been particularly hard hit during the pandemic, negatively affecting their mental health (21). Some studies have shown that women are more likely to lose their jobs permanently and are more concerned about their economic situation than men during the pandemic outbreak (35). Conversely, Hupkau and Petrongolo (36) found that women were more likely to fill jobs that could be performed from home, and they were less affected by the adverse impacts of the pandemic.

Middle-aged adults experienced poor mental health during the pandemic. This finding is in line with previous studies which showed that younger age groups were less satisfied with their lives and had higher rates of depression and anxiety than other age groups during the pandemic (20, 37, 38). The possible explanation for this finding is that young people were more likely to experience job loss and income reduction during the pandemic than older people (21). Moreover, young people in general face a chronically high unemployment rate, which worsened during the pandemic. The pace of entering the labor market during the pandemic period was slow and almost non-existent, indicating their disadvantaged situation in the labor market and the potential deterioration of their mental health, especially those who recently joined the labor market and lost their jobs. Moreover, the increase in age coincides with increased experience and accumulated savings, causing a negative relationship between age and incurring economic hardship, which is expected to translate into a positive relationship between age and mental health during the pandemic. In contrast, other studies showed that the older adults and retirees were more likely to apply home confinement measures, self-isolation, or quarantine, and were, therefore, more likely to suffer from stress, anxiety, and depressive symptoms than younger or working individuals (4).

Our findings also showed that mental health varied significantly by place of residence. Urban residents had worse mental health than their rural counterparts, and this may be due to the pandemic's perceived impact on lifestyle and the labor market in urban areas (34). Moreover, most jobs and various-sized economic activities are concentrated in urban areas and have witnessed high rates of layoffs, reduced working hours, and wage cuts. Consequently, the mental health of urban residents is expected to be worse than that of rural residents. El-Zoghby (26) also highlighted that rural residents reported a lower psychological impact due to the pandemic.

Individuals with low socioeconomic status before the pandemic were more likely to suffer from poor mental health during the pandemic, more vulnerable to the negative effects of job and income losses, and more likely to experience high levels of financial stress, especially with the limited social safety nets. The current study demonstrated that individuals in the high-income quartile had better mental health scores than those in the lower-income quartile, consistent with findings of previous studies that found that income level was positively associated with the ability to maintain well-being (19, 39). Contrary to expectations, individuals with higher education had worse mental health than individuals with less than basic education. This finding is in contrast to earlier findings which found that less-educated individuals were at a higher risk of poor mental health (20, 40, 41). The poor mental health of highly educated individuals may be due to suffering negative labor outcomes during the pandemic as Adams-Prassl et al. (21) demonstrated that educated workers were less likely to lose their jobs compared to less-educated workers.

Previous studies found that the effects of lockdown measures differed among individuals, that is, fathers with children were less affected and more satisfied with their families than fathers without children (5). Having children made individuals more satisfied with their lives than non-parents. Having children alleviated the negative impact of losing jobs and incomes on life satisfaction during the pandemic as parents who lost their jobs have been able to care for their children more than before (8). Recchi et al. (42) found that the COVID-19-related lockdown measures had a positive impact on mental health because they allowed workers to spend more time with family, and the situation was better for those working from home who have kept their jobs and were closer to their families. However, there is no evidence that having children improves the individual's mental health in the current study. The household size also did not have a significant effect on mental health status. In addition, there is no significant evidence that the individual's mental health was adversely affected by marital status as reported by other studies (43), while El-Zoghby (26) found a positive relationship between married respondents and increased financial and home stress during the pandemic.

Unemployment was negatively associated with individuals' subjective well-being. Unemployed individuals had worse mental health than employed during the pandemic. A significant relationship has been widely documented between economic stagnation and poor mental health. Symptoms of depression, anxiety, self-harming behavior, and suicide have increased during and after economic downturns (44). Changes in labor market outcomes were likely to affect individuals' well-being and their mental health status. Some studies found that life satisfaction level was affected more negatively by unemployment among men than women and life satisfaction level among unemployed middle-aged adults was lower than that of other age groups (8, 37). Cotofan et al. (8) emphasized that employment status during the pandemic was a key driver of subjective well-being and the unemployed were less satisfied with their lives than full-time workers during the pandemic. The same is true for inactive people who stopped looking for work during the pandemic. Workers who were unable to work during the pandemic also had lower life satisfaction levels, especially those non-furloughed with income loss (8). Barsoum and Majbouri (13) found that unemployment was negatively correlated with men's well-being, while the burden of housework and childcare was negatively associated with women's subjective well-being. Zivin et al. (45) also found that the economic downturn has adverse impacts on all population segments, but the impacts were worse among underclass groups such as the poor, the unemployed, and the less-educated individuals.

COVID-19 has caused multiple stressors affecting individuals' mental health including self-quarantine, infection concerns, inadequate food supplies, and financial losses (18, 26). Food insecurity was directly associated with poor mental health and psychosocial stressors (46, 47). The decrease in household income was also associated with lower levels of well-being and had negative consequences on the mental health of all household members in Arab countries (13, 20). In line with previous studies, the current study also found that individuals' mental health was negatively affected by experiencing food insecurity and a decline in household income.

The future expectations for income changes differed considerably after the COVID-19 outbreak. Many individuals were more concerned about their economic situation during the prevailing uncertain conditions of the pandemic. Individuals' perceptions were less pessimistic and expected substantial declines in their incomes. Consistent with the literature (41), our findings highlighted that individuals who were very worried about the economic situation experienced substantially lower mental health scores than individuals who were not worried at all. The same is true for individuals who were more worried about contracting COVID-19. Conversely, individuals who adhered to social distancing and wore a mask to prevent infection had higher mental health scores than those who did not.

The Egyptian economy tried to recover by adopting plans to coexist with the pandemic, easing restrictions and removing the daily curfew, resuming international flights, and gradually reopening restaurants, recreational facilities, mass public and private transport, and public and private schools and universities. However, the current study found that mental health status did not improve across waves in line with other studies that found that the adverse effects of the COVID-19 pandemic on mental health are persistent even after the economic recovery (48).

These findings can be drawn upon when designing interventions to help affected individuals during the pandemic. There is a significant gender gap in mental health status. Women were more vulnerable to negative psychosocial outcomes than men and should receive psychosocial support. Women's mental health can be improved by implementing flexible working arrangements, paid sick and parental leave care, and other family-friendly policies. Middle-aged adults, urban residents, highly educated individuals, and poor household members also showed poor mental health, indicating their need for psychological and financial support.

The government should pay special attention to individuals suffering from food insecurity, reduced household income, and job loss. Unemployment benefits and safety nets can play a significant role in improving their mental health. In addition, individuals who have experienced excessive anxiety about the economic situation and fear of contracting the virus should be integrated into psychoeducation programs and other supportive interventions.

Mental health was a major health concern during the ongoing COVID-19 pandemic. Our study contributes significantly to investigating the impact of the pandemic on mental health status. We provided a comprehensive assessment of the factors affecting mental health status in Egypt. Our findings potentially guide healthcare planners and policymakers in making targeted evidence-based decisions to support affected individuals during crises. However, the study has some limitations. First, the sample included mobile users aged 15–64 years. Therefore, results may not be representative of the population because mobile users are often highly educated, men, and at high-income levels (49–51). Second, the survey does not include data to measure the impact of mental health services provided by the government on improving the health status of individuals during the pandemic period. Moreover, the survey does not include any data on the pre-pandemic mental health history. There may be confounding factors due to not addressing the cases suffering mental health problems before COVID-19 or those with a history of psychiatric disorders or treatment.

## 5. Conclusion

The COVID-19 pandemic has disrupted lifestyle and increased depression symptoms and poor mental health. This study examined the relationship between mental well-being and risks, social distancing measures, and other disruptions induced by the pandemic. The findings of the study indicated that worrying about the economic situation and COVID-19 infection, experiencing food insecurity, and household income decline are strong predictors of individuals' mental health. The study also demonstrated that sociodemographic characteristics are contributing factors in shaping mental health status during the pandemic. Women, middle-aged adults, urban residents, unemployed, highly educated individuals, and poor household members are the most vulnerable to poor mental health during the pandemic. Providing counseling and providing mental and social health services to these vulnerable groups are essential.

## Data availability statement

The data analyzed in this study is subject to the following licenses/restrictions: the data are available from The Economic Research Forum (ERF) but restrictions apply to the availability of these data, which were used under license for the current study, and so are not publicly available. To get the micro data, see “OAMDI (29). COVID-19 MENA Monitor Household Survey (CCMMHH), http://www.erfdataportal.com/index.php/catalog. Version 5.0 of the licensed data files; CCMMHH_Nov-2020-Aug-2021. Egypt: Economic Research Forum (ERF).” Requests to access these datasets should be directed to http://www.erfdataportal.com/index.php/catalog.

## Ethics statement

The current study used a secondary data set (29). The data have been collected by the Economic Research Forum (ERF) who maintain all ethics approval and consent to participate. This study is a secondary analysis of an existing data set and it does not deal with any human subject.

## Author contributions

SA-R, FA, EI, and MA: conceptualization, software, and formal analysis. SA-R and MA: methodology. FA, EI, MA, and BK: validation, investigation, and writing—reviewing and editing. FA and MA: resources. SA-R, FA, and MA: writing—original draft preparation. SA-R and MA: visualization. BK: supervision. SA-R, FA, EI, MA, and BK: project administration. FA, EI, and MA: funding acquisition. All authors have read and agreed to the published version of the manuscript.
