# Supplementary material for: Predictors of mental health problems during the COVID-19 outbreak in Egypt in 2021
Source: Front Public Health. 2023 Nov 9;11:1234201. doi: 10.3389/fpubh.2023.1234201 (PMC10665964; doi:10.3389/fpubh.2023.1234201)
Supplement: Supplementary file 1 [file Data_Sheet_1.docx]

**Appendix**

**Table A**. Percentage of respondents reported frequencies for their various feelings during the pandemic

|  | **At no time** | **Some of the time** | **Less than half of the time** | **More than half of the time** | **Most of the time** | **All the time** |
| --- | --- | --- | --- | --- | --- | --- |
| **Feeling cheerful and in good spirits** | 21.9% | 37.2% | 15.5% | 6.9% | 11.3% | 7.1% |
| **Feeling calm and relaxed** | 18.3% | 39.1% | 19.2% | 7.3% | 9.3% | 6.8% |
| **Feeling active and vigorous** | 12.9% | 33.3% | 19.8% | 11.3% | 10.5% | 12.1% |
| **Feeling refreshed and rested upon waking** | 16.9% | 35.9% | 21.8% | 7.8% | 9.8% | 7.8% |
| **Daily life is full of things that matter** | 5.7% | 27.4% | 17.1% | 11.2% | 13.3% | 25.4% |


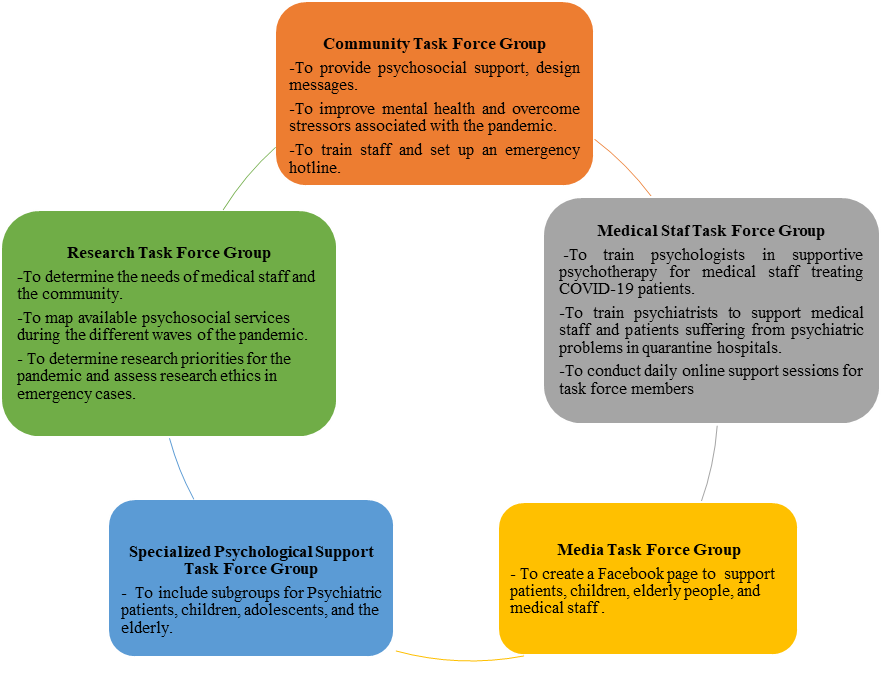


Fig A.1. The functions of the five task force groups during the pandemic
